# Connecting the Dots: The Interplay Between Stroke and the Gut-Brain Axis

**DOI:** 10.7759/cureus.37324

**Published:** 2023-04-09

**Authors:** Pooja M Murthy, Jayashankar CA, Venkataramana Kandi, Mithun K Reddy, Ganaraja V Harikrishna, Kavitha Reddy, Ramya JP, Ankush N Reddy, Jigya Narang

**Affiliations:** 1 Medicine, Vydehi Institute of Medical Sciences and Research Centre, Bangalore, IND; 2 Internal Medicine, Vydehi Institute of Medical Sciences and Research Centre, Bangalore, IND; 3 Clinical Microbiology, Prathima Institute of Medical Sciences, Karimnagar, IND; 4 Neurology, Vydehi Institute of Medical Sciences and Research Centre, Bangalore, IND; 5 General Medicine, Vydehi Institute of Medical Sciences and Research Centre, Bangalore, IND

**Keywords:** therapeutic approach, stroke, dysbiosis, gut-brain axis, gastrointestinal tract

## Abstract

This article discusses the interplay between the gut-brain axis and stroke, a multifaceted neurological disorder that affects millions of people worldwide. The gut-brain axis is a bidirectional communication network linking the central nervous system (CNS) to the gastrointestinal tract (GIT), including the enteric nervous system (ENS), vagus nerve, and gut microbiota. Dysbiosis in the gut microbiota, alterations in the ENS and vagus nerve, and gut motility changes have been linked to increased inflammation and oxidative stress, which are contributing factors in the development and progression of stroke. Research on animals has shown that modifying the gut microbiota can impact the results of a stroke. Germ-free mice displayed improved neurological function and decreased infarct volumes, indicating a positive effect. Furthermore, studies in stroke patients have shown alterations in the gut microbiota composition, indicating that targeting dysbiosis could be a potential therapeutic strategy for stroke. The review suggests that targeting the gut-brain axis may represent a potential therapeutic approach to reduce the morbidity and mortality associated with stroke.

## Introduction and background

Stroke is a multifaceted neurological disorder and a major cause of mortality and disability worldwide, affecting millions of people annually [1]. Stroke may be classified into two types: ischemic stroke and hemorrhagic stroke. Ischemic stroke is the predominant type and generally results from a clot that essentially limits oxygen and nutrition supply to the brain and results in damage [2]. The available research suggests that multiple mechanisms are involved in ischemic-type brain injury and damage. However, limited therapeutic approaches like thrombectomy are available for better patient management [3]. Tissue-type plasminogen activator (tPA) has been recently approved by the United States Food and Drug Administration (USFDA) for the treatment of acute ischemic stroke [2,3]. The major drawback related to drug therapy in treating brain injury is the complexity associated with drug delivery. It has been noted that the blood-brain barrier (BBB) and blood-cerebrospinal fluid barrier and brain vasculature contribute to difficulties in drug delivery [2]. The application of nano warriors, genomic deletion of endothelial microRNA-15a/16-1, and repurposing metformin among other strategies have been investigated for the treatment of stroke [4-6]. Despite advancements in the management of stroke, there is a significant need for novel therapeutic approaches to improve patient outcomes.

The gut-brain axis is a bidirectional communication network linking the central nervous system (CNS) to the gastrointestinal tract (GIT), including the enteric nervous system (ENS), vagus nerve, and gut microbiota. Emerging evidence implicates the gut-brain axis in the pathophysiology of various neurological disorders, including stroke. The ENS is a complex network of neurons and glial cells that innervates the GIT and functions independently of the CNS. The vagus nerve connects the brainstem to the GI tract and transmits information about the state of the gut to the CNS, regulating GIT function. The gut microbiota can also communicate with the CNS through the production of neurotransmitters and immune modulation [7]. In this review, we intend to provide an overview of the interplay between the gut-brain axis and stroke and the gut-brain axis's potential as a therapeutic target, including the administration of prebiotics, probiotics, and synbiotics, fecal microbiota transplantation (FMT), and modulating the vagus nerve.

## Review

Anatomy and physiology of the gut-brain axis

The gut-brain axis is a complex system involving interactions between the GIT and the CNS through the ENS, the vagus nerve, and the gut microbiota. The ENS, also known as the "second brain," is a network of neurons and glial cells that operates independently of the CNS, and regulates GI motility and local immune responses through the myenteric and submucosal plexuses. The vagus nerve connects the brainstem to the GIT and regulates intestinal motility, secretion, inflammation, and sensory information transmission. The gut microbiota, a community of microorganisms living in the GIT, communicates with the CNS through neurotransmitters and immune modulation. Together, these systems maintain gut homeostasis and transmit information to the CNS about the state of the gut [7].

Gut-brain axis and stroke

Stroke is a major cause of mortality and morbidity worldwide, and it is characterized by the disruption in blood supply that leads to a sudden loss of brain function. Ischemic stroke is the most prevalent form of stroke and is caused by a blockage in a blood vessel supplying the brain. On the other hand, hemorrhagic stroke is caused by bleeding in the brain [8].

Mechanisms of Stroke

The pathophysiology of stroke is a multifaceted and intricate process that involves numerous cellular and molecular mechanisms such as inflammation, oxidative stress, excitotoxicity, and apoptosis. These mechanisms can result in neuronal damage and death, as well as disturbances in the blood-brain barrier and changes in cerebral blood flow [9].

The Function of the Gut Microbiota in Stroke

Recent research has suggested that the gut microbiota plays a crucial role in the pathophysiology of stroke by regulating the immune system. An imbalance in the gut microbiota composition, known as dysbiosis, has been linked to increased inflammation and oxidative stress, which are contributing factors in the development and progression of stroke. Animal studies have demonstrated that altering the gut microbiota can affect the outcome of stroke, with germ-free mice exhibiting better neurological function and reduced infarct volumes. Furthermore, studies in stroke patients have shown alterations in the gut microbiota composition, indicating that targeting dysbiosis could be a potential therapeutic strategy for stroke [10].

Role of the ENS and Vagus Nerve in Stroke

The ENS and vagus nerve have been identified as significant factors in the pathogenesis of stroke. The ENS is responsible for controlling intestinal blood flow and immune responses and is thought to be involved in the regulation of systemic inflammation. The vagus nerve, which acts as the primary communication pathway between the CNS and ENS, has been found to have neuroprotective effects in animal models of stroke. By reducing inflammation and oxidative stress, stimulation of the vagus nerve has been shown to decrease infarct volume and enhance neurological function [11].

Effect of Stroke on Gut Function

Stroke can affect gut function in various ways. Dysphagia, or difficulty in swallowing, is a common stroke complication that can lead to pneumonia and other adverse outcomes [12]. Furthermore, stroke can cause changes in gut motility and microbiota composition, which can contribute to complications like infections and malnutrition after stroke [13]. The gut-brain axis is believed to play a critical role in stroke pathophysiology, and hence targeting it may represent a potential therapeutic approach to reduce the morbidity and mortality associated with stroke [14].

Animal models of stroke and the gut-brain axis

Animal models have been extensively utilized to understand the mechanisms and potential treatments for stroke. In recent times, these models have also been employed to explore the role of the gut-brain axis in stroke [14].

Studies Using Rodents to Investigate the Gut-Brain Axis in Stroke

In order to investigate the impact of stroke on the gut-brain axis, several studies have employed rodent models of stroke. For instance, an experiment conducted on rats revealed an increase in the abundance of potentially pathogenic bacteria in the gut microbiota composition after an induced stroke. Similarly, another study demonstrated changes in the expression of genes related to the ENS in the colon of mice following stroke [15].

Findings on the Impact of Stroke on the Gut Microbiota and ENS in Animal Models

Animal models of stroke have helped researchers understand the connection between stroke and the gut-brain axis [16]. Studies have revealed that stroke-induced changes in the gut microbiota can cause inflammation and oxidative stress, which can worsen brain damage. Stroke may also lead to gut dysmotility and dysphagia through alterations in the ENS [17].

The Potential Use of Animal Models in Developing New Therapies for Stroke

Animal models of stroke have provided important information regarding potential therapeutic targets for stroke treatment. In particular, rodent studies have demonstrated that modulating the gut microbiota through probiotics or FMT can improve stroke outcomes [18]. Additionally, stimulation of the vagus nerve has shown promise as a neuroprotective strategy in animal models of stroke [19]. These findings offer valuable insights into the role of the gut-brain axis in stroke and may lead to the development of new treatments for stroke-related complications.

Clinical studies on the gut-brain axis in stroke

Clinical investigations have also explored the gut-brain axis in stroke patients, in addition to animal studies. These studies have focused on the effects of stroke on the gut microbiota and the potential of gut microbiota modulation as a therapeutic strategy for stroke.

Evidence for Alterations in the Gut Microbiota in Stroke Patients

Studies conducted on stroke patients have demonstrated changes in the composition of their gut microbiota. For instance, one study has revealed that stroke patients had reduced microbial diversity and increased potentially harmful bacteria compared to healthy individuals [20]. Another study has found that stroke patients had lower levels of beneficial bacteria such as *Bifidobacterium *and *Lactobacillus *and higher levels of pro-inflammatory bacteria like *Enterobacteriaceae *[21]. It has been observed that stroke patients have lower bacterial diversity and decreased bacterial abundance compared to healthy individuals. Several specific bacterial taxa are also found to be altered in stroke patients. For instance, the abundance of *Prevotella*, a genus of gram-negative bacteria known to produce short-chain fatty acids, is significantly decreased in stroke patients. Conversely, *Bacteroides*, a genus of gram-negative bacteria associated with inflammation, is significantly increased in stroke patients [22]. The gut microbiota composition in healthy individuals clearly differs from stroke patients and people with chronic health conditions like cardiovascular diseases. Dysbiosis is uncommon among people who do not consume antibiotics or those who had undergone any major surgeries in the recent past [23-25]. The differences in gut microbiota composition between healthy individuals and stroke patients are depicted in Table 1.

**Table 1:** The differences in gut microbiota composition between healthy individuals and stroke patients

Gut microbial composition	Healthy individuals	Stroke patients
Bacterial diversity	High	Low
Bacterial abundance	Stable	Decreased
*Prevotella *abundance	High	Low
*Bacteroides *abundance	Low	High
*Akkermansia *abundance	Low	High
*Lactobacillus *abundance	High	Low
Fecal bacterial abundance	High	Low
*Clostridium *abundance	Low	High

The Relationship Between Stroke Severity and Gut Dysbiosis

The relationship between stroke severity and gut dysbiosis has also been examined in several studies. Results of a previous study revealed that stroke patients with more severe neurological impairments had a greater abundance of potentially pathogenic bacteria in their gut microbiota [26]. Another study demonstrated that the severity of gut dysbiosis was linked to worse outcomes in stroke patients, including increased mortality and disability [27].

The Potential for Targeting the Gut Microbiota as a Therapeutic Approach to Stroke Management

**Figure 1:**
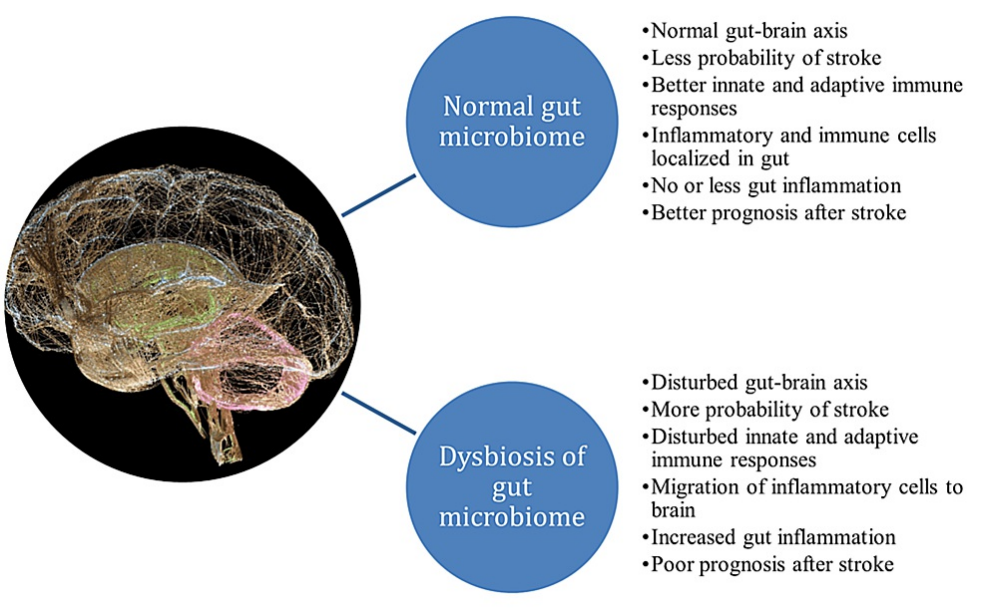
Pictorial representation of the role of gut microbial flora in the development of stroke and its management This Figure has been created by the authors

Clinical studies have demonstrated alterations in the gut microbiota in stroke patients, leading to increasing interest in targeting the gut microbiota as a therapeutic approach [21]. Clinical trials investigating probiotics and FMT have shown promising results in stroke patients, with probiotics leading to significant improvements in the National Institutes of Health Stroke Scale (NIHSS) score and reduced inflammation, while FMT led to improved gut dysbiosis and neurological function [27]. These findings suggest that targeting the gut microbiota may be an effective strategy for treating stroke-related complications. A Pictorial representation of gut microbial flora and its role in the development of stroke and its management is depicted in Figure 1.

Therapeutic interventions targeting the gut-brain axis in stroke

The evidence for changes in the gut-brain axis in stroke has led to a growing interest in developing therapeutic interventions that target this axis. Among the interventions that have been explored are prebiotics, probiotics, synbiotics, FMT, and modulation of the vagus nerve.

Prebiotics, Probiotics, and Synbiotics

Prebiotics, probiotics, and synbiotics have been studied as therapeutic interventions that target the gut-brain axis in stroke patients. Prebiotics are non-digestible food components that stimulate the growth of beneficial bacteria in the gut, while probiotics are live microorganisms that confer health benefits when consumed in adequate amounts. Synbiotics are a combination of prebiotics and probiotics that work together to promote the growth of beneficial bacteria in the gut [28]. Clinical studies have shown that the administration of probiotics containing *Lactobacilli *and *Bifidobacterium *strains improved neurological function in stroke patients, and the administration of a synbiotic containing *Bifidobacterium *and oligofructose improved cognitive function in stroke patients [29].

Fecal Microbiota Transplantation

The transfer of fecal matter from a healthy donor to a patient suffering from dysbiosis associated with a disease is known as FMT [30]. FMT has demonstrated efficacy in the management of recurrent *Clostridioides difficile* infection, and it is presently being studied as a prospective treatment strategy for stroke [31]. A recent study found that FMT in stroke patients was associated with improvements in gut microbiota diversity and stroke outcomes [24]. However, larger randomized controlled trials are necessary to confirm these findings and establish the optimal timing and dosage of FMT in stroke patients.

Modulating the Vagus Nerve

Modulating the vagus nerve has been studied as a potential therapeutic approach in stroke, as it is a critical element of the gut-brain axis. Previous studies have shown that stimulation of the vagus nerve improved stroke outcomes in rats [32]. Currently, a clinical trial is underway to investigate the effectiveness of vagus nerve stimulation in human stroke patients. The outcome of this trial will provide significant insights into the potential of using vagus nerve stimulation as a therapeutic approach to stroke management [33].

Novel Interventions for Targeting the Gut-Brain Axis in Stroke

Researchers are exploring novel interventions to target the gut-brain axis in stroke, such as using stem cell-derived exosomes to modulate the gut microbiota and improve outcomes [34]. Other potential interventions include targeting gut microbial metabolites and developing microbiome-targeted drugs [35]. These interventions show promise for treating stroke-related complications, but more research is needed to determine the optimal approach and timing for their use in stroke patients.

Drawbacks of present research studies

Despite the increasing evidence supporting the role of the gut-brain axis in stroke, there are several limitations in the current research. One of the major limitations is the lack of standardization in experimental protocols and study design, which makes it difficult to compare results across studies. Small sample sizes in some studies may limit the generalizability of findings. In addition, many studies have been conducted using animal models, and the findings may not be directly applicable to humans [36].

Moreover, establishing a causal relationship between gut dysbiosis and stroke can be challenging due to the complex and dynamic nature of the gut microbiota and multiple known vascular risk factors could individually or in combination may predispose to an ischemic stroke. It is difficult to determine whether alterations in the microbiota are a cause or a consequence of stroke. Therefore, further research is needed to overcome these limitations and provide a better understanding of the role of the gut-brain axis in stroke [37].

Future research directions

There is still great potential for further research on the gut-brain axis in stroke, despite the limitations outlined above. Personalized medicine approaches based on gut microbiota and ENS function are promising areas of research. By combining microbiome analysis, neural imaging, and clinical assessments, it may be possible to identify patient-specific factors that contribute to stroke risk and tailor treatments accordingly. Additionally, there is a need to develop novel therapeutic interventions targeting the gut-brain axis in stroke. While prebiotics, probiotics, and FMT have shown some promise in animal models, their efficacy and safety in human patients require further investigation. Moreover, there may be other unexplored interventions that could prove beneficial [38].

In a very recent report, it was observed that immune responses could play a crucial role in the cause and effect of brain function/damage. Especially, after an ischemic stroke, the prognosis depends on the gut microbiome and its influence on immune regulation that could enable the control of damage to brain cells. Additionally, the consumption of probiotics and gut microbial transplantation can improve post-stroke prognosis [39].

Intestinal microbial colonization and its alteration during aging, stroke, and other conditions require further understanding. This is evident by the occurrence of *Clostridium perfringens* among healthy persons. Alternatively, *Streptococcus *species were abundant among the unhealthy elder population. *Aspergillus *and *Escherichia coli *were found in higher numbers among stroke patients [40-42].

## Conclusions

Dysbiosis in the gut microbial composition may influence stroke severity and its prognosis. Therapeutic interventions such as prebiotics, probiotics, synbiotics, FMT, and vagus nerve modulation have shown promise in animal models and clinical studies. Despite limitations in the current research, future studies may help refine our understanding of the gut-brain axis in stroke and lead to the development of new treatments in the management of stroke. Personalized medicine approaches based on gut microbiota and ENS function may provide tailored treatments for stroke patients. The gut-brain axis is a promising area of research with the potential to prevent stroke and significantly improve patient outcomes.
